# What are “good outcomes” for adolescents in public mental health settings?

**DOI:** 10.1186/s13033-018-0183-5

**Published:** 2018-01-19

**Authors:** Kristina O. Lavik, Marius Veseth, Helga Frøysa, Per-Einar Binder, Christian Moltu

**Affiliations:** 1 Department of Psychiatry, District General Hospital of Førde, Førde, Norway; 2 Department of Clinical Psychology, University of Bergen, Bergen, Norway; 3 Department of Community Mental Health Service, Askøy kommune, Bergen, Norway

**Keywords:** Routine outcome monitoring, Clinical feedback systems, Outcome, Recovery, User involvement research, Youth mental health services

## Abstract

**Background:**

In line with the evidence-based paradigm, routine outcome monitoring and clinical feedback systems are now being recommended and implemented in youth mental health services. However, what constitutes a good outcome for young service users is not fully understood. In order to successfully monitor outcomes that are clinically and personally relevant for the service user that are to benefit from these systems, we need to gain more knowledge of what young service users value as meaningful outcomes of youth mental health services.

**Aim:**

To contribute knowledge into what constitutes “good outcomes” from the experiences of adolescent service users in public mental health systems.

**Methods:**

A qualitative in-depth study of the experiences and reflections from 22 adolescents aged 14–19 years, currently or recently being in public mental health services. The data material was analyzed using a systematic step-wise consensual qualitative research framework for team-based analysis.

**Results:**

An overarching theme of outcome as having developed a stronger autonomy and safer identity emerged from the analysis, with the subsequent five constituent themes, named from the words of the adolescent clients: (1) I’ve discovered and given names to my emotions, (2) I’ve started to become the person that I truly am, (3) I’ve dared to open up and feel connected to others, (4) I’ve started saying yes where I used to say no, and, (5) I’ve learned how to cope with challenges in life.

**Conclusion:**

“Good outcomes” in youth mental health services should be understood as recovery oriented, sensitive to developmental phases, and based on the personal goals and values of each adolescent client.

## Background

What do adolescents in mental health services experience as the most important topics to give feedback about when outcome is systematically monitored? In line with the evidence-based practice paradigm, routine outcome monitoring (ROM) and clinical feedback systems (CFS) are now being recommended internationally in youth mental health settings [1]. ROM and CFS systems enable clinicians to track the client’s response to treatment throughout episodes of care and enhance user involvement in form of client feedback. Research indicates that youths improve faster when their clinicians are provided with ongoing outcome feedback compared to peers treated at clinics without such systems [2]. Furthermore, young service-users are generally positively inclined towards the use of outcome measures in mental health services [3], if these are seen as relevant to them.

However, if clinicians are to routinely track treatment outcomes and use them constructively to improve the effectiveness of mental health services, some important questions need to be raised: What are “good” outcomes in youth mental health settings? For whom, and by whom, are outcomes defined? How do we ensure that clinicians are tracking outcomes that are relevant for adolescent service-users?

Similar questions have been researched for psychological treatments with adults. Results underscore that how one comes to function in important social roles [4], how relationally safe and able to set constructive boundaries one becomes [5, 6], how accepting and understanding of own affect and experiences one manages to be [7], and how much insight and understanding into mental health needs and patterns one develops [5, 6], are all important parts of recovery and good outcomes when studied from the adult client’s perspective. Symptom reduction is one domain that is part of clients’ understanding of a good outcome, but research has tended to over-emphasize this single domain over the very important others [5, 6]. However, one cannot conclude that what are relevant outcomes for adults is mirrored when studying the adolescents’ perspectives. Young service-users live in a different social context than adults do, and certain outcomes of psychotherapy will also possibly be prioritized by them due to the developmental tasks and challenges of adolescence. We recognize a lack of knowledge into adolescent perspectives.

Although user involvement has become central to public health care policies, less effort has been spent on translating user-defined outcomes into mental health practice [8, 9]. This is particularly problematic as service-users tend to value different aspects of their mental health and social functioning than mental health care professionals. For example, clients put less emphasis on symptom reduction than they do on improvement in other life arenas [10, 11]. In consequence, clinicians risk monitoring outcomes that are not fully meaningful and important from a user perspective. In youth mental health settings, it appears that standardized clinical outcomes match poorly with young service-users own conceptualizations [10]. Young service-users tend to emphasize increased emotion regulation, self-awareness and connectedness in social relationships as important treatment outcomes [12], aspects which are seldom included in standardized measures. Furthermore, most outcome measurement scales have an exclusive focus on reduction of symptoms, and are therefore rarely capturing aspects of personal recovery beyond clinical improvement [11].

Monitoring changes in the health and wellbeing of young service-users should be beneficial to all stakeholders: to the adolescents themselves, their families, the treatment providers, and policy makers. Yet, if changes obtained in psychotherapy are to matter in real life settings, “good outcomes” need to be defined by young service-users, as most would argue that they hold significant answers to what a meaningful life comprises for them. A sustained effort in involving young-service users collaboratively from the development of measures, to their application in clinical settings is needed to accomplish this [13].

Research demonstrates that public mental health services, including psychotherapeutic interventions, have challenges when it comes to drop-out and disengagement [14]. This is particularly true for young service users [15, 16]. An important step toward engaging adolescents in their ongoing mental health treatment more successfully is to build mental health care systems that relate better to their needs and perspectives on what constitutes good outcomes. Given the absence of young service-users’ voices in the literature concerning ROM and CFS [13], we found it necessary and fruitful to investigate the following research question: What are meaningful and good outcomes in youth mental health settings as experienced by adolescents in ongoing or recently ended mental health treatment?

## Methods

### Setting

This study was organized and financed by Førde Health Trust, a public hospital in the western region of Norway. Five public regional outpatient clinics participated in the project. Norway has a single payer public health care system, and the participating clinics provide specialized mental health services to children, adolescents and families free of charge, in line with national standards and guidelines for evidence based practice. The staff consists of a variety of mental health professionals, such as psychiatrists, clinical psychologists and trained psychotherapists. In terms of offered psychotherapies, both eclectic approaches, systemic/family therapy and structured models (such as for example CBT and MBT) are utilized, depending upon the background of the clinician and the needs and preferences of each individual adolescent.

The interviews were semi-structured, and the adolescents were asked to elaborate on the following open-ended questions: (1) In your opinion, what is it to “get better”? (2) How can we know if an adolescent is getting better as result of mental health services? (3) When reflecting upon own experiences with mental health services, what may have helped you get better? (4) What may have been unhelpful? Each question was intended to open for exploring the adolescents’ relevant experiences, and we had developed follow-up prompts and strategies to help the interview toward the personal and concrete level of talk, such as “can you remember a specific experience that stood out as important?” and “can you think of an example where you noticed this?”.

### Participants

Participants were recruited using a convenience sample strategy. Participating clinics committed to inviting at least three young clients to the study, where the clinicians at participating clinics agreed to inform the clients currently in treatment verbally about the purpose and design of the study. Clients who were interested in participation were provided with an invitational letter containing details of the study and contact information to the researchers.

As we aimed to explore the experiences and opinions of young clients openly, we established a wide inclusion criterion: any young client between 12 and 19 years of age, who was in ongoing or recently ended psychotherapy, and who had an interest and desire to contribute to the project, was welcome. Clinicians were instructed to invite patients whom they considered as “experienced service-users, defined as having participated in psychological treatment for at least 6 months. However, as a result of the sample strategy, numbers of how many patients who decided to decline the invitation was not gathered systematically. Clients got to choose whether they wanted to participate in an individual interview or in a focus group interview. To ensure participant safety we established one exclusion criteria: clients with an active psychosis or mania at the time of the study were not to be invited to the study. A psychotic or bipolar diagnosis was not an exclusion criterion if the client was in a stable non-psychotic or non-manic phase.

The resulting sample consisted of 22 young clients aged 14–19 years: 16 girls (mean age 16, *SD* 1.4), and six boys (mean age 17, *SD* 1.5), all Norwegians by birth. Two participants had reached age 19 at the onset of the interviews, and were included because both had recently completed therapy. In total, six participated in individual interviews, whereas the remaining contributed in four different focus groups ranging from five to two participants per group. Although we aimed at four participants as a minimum, only two attended to one of the focus groups due to dropout and recruitment difficulties. The groups were organized based on location, meaning that all participants in one group belonged to the same outpatient clinic. Five of the adolescent participants belonged to a reference focus group from a service user organization, all having current or recent (within 1 year) experience from mental health services. As members of a mental health user organization, they travelled nationally and gave talks about own personal experiences as young service users, hereby providing advise as “mental health pro’s” to clinicians, mental health systems and policy makers. They were invited to share their opinions, experiences and thoughts on the same line as the remaining adolescent participants, meaning that the data material originating from this one focus group were analyzed and emphasized in the same manner as the rest of the data.

Duration of mental health problems and diagnostic data were not collected, as this study aimed to investigate the research questions trans-diagnostically. However, most clients chose to openly disclose some details about their mental health problems. As a group they were heterogeneous, and they appeared to mainly struggle with anxiety, depression, relational trauma, self-harm behavior and suicidal ideation. Several of the clients reported having mental health problems since early childhood resulting in a long history with both hospitalization and out-patient treatment in mental health and child care systems. None of the participants were newly referred to treatment, meaning that the participants had at least 6 months of experience as a client undergoing mental health treatment. Several had experience with more than one therapist in psychotherapeutic settings. Preliminary analyses were carried out throughout the data collection phase. By participant 18 we experienced that additional data did not add substantial new aspects to the analyses. After participant 20 we therefore established that data saturation was reached, and ended the data collection phase.

### Researchers

KOL is a clinical psychologist at District General Hospital of Førde, Norway. HF is a clinical psychologist at Askøy Community Mental Health Service, Norway. PEB is a professor of clinical psychology at the Department of Clinical Psychology, University of Bergen, Norway, and a clinician with 22 years of psychotherapy experience. MV is a clinical psychologist with 8 years of experience and associate professor at the Department of Clinical Psychology, University of Bergen, Norway. CM is a clinical psychologist with 10 years of experience, chief advisor at District General Hospital of Førde, Norway, and a professor of clinical psychology at Department of Health Studies, Western Norway University of Applied Science. Our interest for the research question arose from a shared fundamental humanistic, integrative and relationally oriented standpoint. We all place high value in real user involvement as an enriching and integral part of research and clinical work.

### Data collection method

Focus groups were the main strategy for data collection. In focus groups participants are allowed to build on and develop each other’s understandings, resulting in potentially deeper and more elaborative data [17–19]. However, participating in a focus group may be anxiety provoking for some adolescents. We found it important to let the adolescents voice their opinions and experiences in an environment that felt safe to them, and, therefore, we also offered individual interviews to all participants. Individual interviews are especially well suited for in-depth and detailed exploration of lived experiences [20, 21]. Six adolescents chose this option.

Two interview guides were developed for the different interview settings. Although worded differently to mirror the respective interview formats, they were similar in content. The interviews were semi-structured, and we strived to balance structure and flexible openness to whatever came up during the conversation.

KOL moderated two focus groups, co-moderated one focus group and conducted five individual interviews. HF moderated one focus group and conducted one individual interview. CM co-moderated one focus group. In sum this study builds on 6 individual and 4 focus group interviews lasting from 40 min to 2 h. The duration of individual interviews were generally one to one-and-a-half hour and 2 h for the focus groups. All interviews were audio-recorded and transcribed verbatim in Norwegian language for analyses.

### Data analysis

Data was analyzed using a systematic step-wise consensual qualitative research framework for team-based analysis [22, 23]. We proceeded as follows: (1) KOL, HF and CM read all the transcripts closely to get familiar with the data material, and made associative notes of emerging tentative themes, (2) KOL, HF and CM met at a 1-day analysis seminar and developed consensually a preliminary thematic structure, (3) after the analysis seminar, KOL re-read in-depth with the preliminary structure in mind to control the correspondence, (4) the thematic structure with illustrative quotes was presented to MV, and to PEB who has a longstanding experience as an adolescent clinician and researcher, and who functioned as a critical auditor. Illustrative quotes and naming of the themes were translated into English language after the data analysis was completed.

### Ethical considerations

This project was sent to the Norwegian Regional Ethic Committees for consideration and was formally exempted from a full consideration. Due to Norwegian regulations, parents of clients aged 15 or younger received, approved and signed an informed consent on behalf of their children prior to participation. Clients aged 16 and older received verbal and written information, and signed an informed consent prior to participation. One client had social interaction difficulties and was provided with a list of questions a week before the interview in order to prepare. We also provided individual interviews as an option for all adolescents that wanted to participate. The adolescents were asked to talk from own concrete experiences with suffering and recovery. We were highly aware about this during preparation phases and during the interviews, and strived to encounter them with an open, non-judgmental attitude. A small debriefing was also done after the interview, and all clients got followed-up in therapy, with some participants from the reference focus group as an exception. One participant received help from the interviewer to change therapist after the interview.

## Findings

Positive outcome experiences were formulated as achieving a sense of a stronger autonomy and safer identity. Thus, good outcomes were understood as ongoing processes towards the life they wanted to live.

**Fig. 1:**
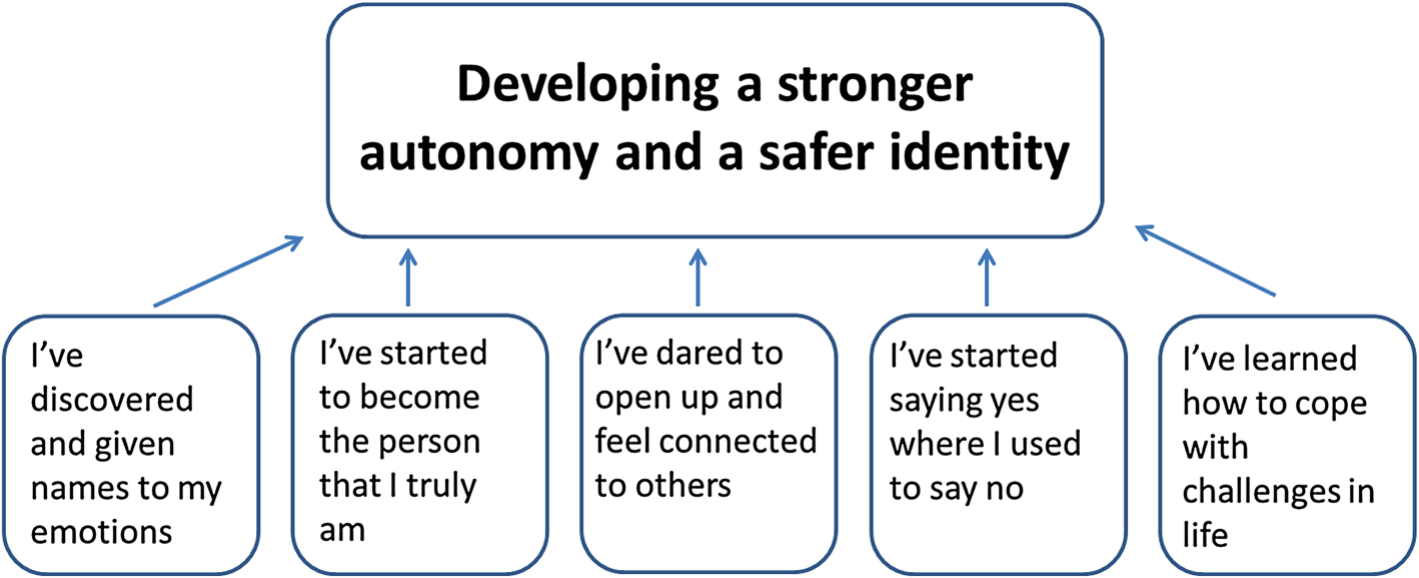
Illustration of the overarching theme and related sub-themes

This overarching theme is reflected through six sub-themes representing nuances and variation in what constitutes a good outcome, named from the words of the adolescent clients: (1) having unpacked and discovered emotions, (2) having started to become the person that I truly am, (3) having dared to open up and feeling connected with others, (4) having started saying yes to new challenges in life, and (5) having learned how to cope with challenges in life (see Fig. 1).

An overview of the representativeness of each theme can be found in Table 1. As can be read from the table, four of the five themes are considered to have broad coverage across participants, meaning that they were present in all or all but one interview. The theme “I’ve discovered and given names to my emotions” had less coverage across participants, being present in seven out of 11 interviews, In the following we detail each of the themes.

**Table 1:** Overview of the different themes and their representativeness in the interviews

	I’ve discovered and given names to my emotions	I’ve started to become the person that I truly am	I’ve dared to open up and feel connected with others	I’ve started saying yes where I used to say no	I’ve learned to cope with challenges in life
Participant 1	•	•	•	•	•
Participant 2		•	•	•	•
Participant 3		•	•	•	•
Participant 4	•		•	•	•
Participant 5	•	•	•	•	•
Participant 6		•	•	•	•
Participant 7	•	•	•	•	•
Focus group 1	•	•	•	•	•
Focus group 2	•	•	•	•	•
Focus group 3		•	•	•	•
Focus group 4^a^	•	•	•		•

^a^Reference focus group

### I’ve discovered and given name to my emotions

Emotional work was mentioned as an important aspect of the therapy process. Several of the adolescents talked about their previous deficiency in recognizing, describing and understanding own emotional reactions, and how their difficulties with handling and communicating intense emotions often led to problems in their social and inner life. In collaboration with the therapist they had opened “black boxes” hidden in their mind and together explored their content, learning that behind a chaos of undefined emotions they were, for example, scared, lonely or angry. Thus, getting better was learning how emotions operated in the body and understanding what was happening on the inside. For example, the following quote from one participant illustrates how getting better was facilitated when the therapist helped her getting aware and communicating what she was feeling:

I didn’t know the difference between… different emotions. I have two emotions, and that’s angry and… well, actually, it’s happy and not happy. […] “Can you please describe to me how you’re feeling?” So I feel this is how a psychologist can help me. If I’ve like experienced something that actually made me sad or if my pet died or if I’d broke up with my boyfriend, that’s sad. If something stupid happened and I got laughed at, I’d feel angry. That sort of stuff. That they [therapists] teach us, people that don’t recognize different emotions, that they teach us the different emotions (girl, 17 years old, focus group 4).

Furthermore, having learned about own emotional reactions became important to find directions in their lives. It became clearer to them how emotions are linked to needs. When something painful happened, for example if a boyfriend broke up or a pet died, they had learned to self-soothe and fulfill own essential needs by seeking comfort and support from others. They also had become more aware of their embodied reactions, understanding that nausea, headaches or trouble sleeping were tied to their feelings. How getting better could manifest in the body is illustrated in the following quote:

If you’re not fine it affects everything in the body. So, when the stress level gets lowered you get better. Take me as an example, I had terrible sleep quality and woke up stiff as a stick. [careful laughter] In the worst periods I threw up during the nights. And I’ve made some changes now on high school, and my sleep isn’t perfect but I don’t throw up anymore (girl, 16 years old, individual interview 5).

Altogether, positive outcome experiences—or aspects of “getting better”– were formulated as having learned how to handle intense emotional reactions and fulfilling own basic needs. As an example, one positive outcome was when emotional work in therapy manifested as a feeling of being able to breathe and relax. In their own words, getting better was the feeling one got when nausea, headache and tensions let go, and to no longer be trapped in an overwhelming emotional chaos.

### I’ve started to become the person that I truly am

Most adolescents were concerned about the pressure placed on today’s generation of youth. They felt compelled to look, feel and act in certain ways to meet an unreachable standard. In order to feel successful one had to perform at all kinds of arenas at the same time, such as for example education, work, and appearance. When adolescents talked about getting better, this was linked to having broken themselves freer from the demands of society, school, parents or friends. For many of them, getting better was about becoming the person that they truly felt they were or wanted to be. One participant put it this way:

You need to get to the core of your problems. What’s underneath it all. Many today speak of *generation performance* where a lot of youths struggle with feelings of inferiority which drives it all. It’s there, chasing confirmation and acceptance, or good grades, because you aren’t able to settle with yourself*. That I am me.* And it doesn’t matter if I get a D, right? It is not *that* that’s going to define who I am (girl, 16 years old, focus group 2).

In addition to stop conforming to the perceived norms and demands of society, the adolescents also stressed that getting better was to establish an identity that did not equal having a psychiatric diagnosis. Thus, getting better was to separate one’s identity and one’s problems, as illustrated by these two participants:

I want to become the person I feel that I truly am. For me getting better is to feel that I am *myself*. That I am not anxiety. Rather, that *I am me.* [laughs a little] And that’s really… I don’t need to have, like, a job, education, friends, as long as I feel that *I am in control* of my head, my thoughts, and not my disease, in a way (girl, 16 years old, focus group 3).

This might sound very weird. But to give up on… my own… it is hard to explain… but, to give up on my own identification with my inner pain. I’ve had so many problems so long that I’d started to believe that I was… that it was my identity (boy, 19 years old, focus group 4).

Self-esteem and self-regard was also an aspect of this as learning to know and accept oneself through therapy had made it easier to take pride in oneself. This quote may illustrate:

I learned to know myself at a very, very young age [as a result of therapy]. And that has helped me as I have grown older. People often tell me that it seems that I… kind of… know who I am, really (girl, 17 years old, individual interview 6).

Another participant told how he had started living by the motto: “What would a person who loved himself do?” (boy, 19 years old, focus group 4), and began to express his feelings through writing and playing the piano. Other adolescents confirmed this, telling how doing hobbies was a way of exercising ones’ identity and the deliberating feeling of actually being the person they were without feeling ashamed. Getting better was in that way trusting and exercising ones true and self-chosen identity.

### I’ve dared to open up and feel connected with others

Although positive outcomes were referred to as a chain of inner processes towards greater emotional- and self-awareness, at some point one needed to turn “inside-out” and eventually dare to relate deeply with meaningful others. One participant described how mental health problems could make you reserved and uncommunicative:

When you’re in great pain or if you have… some sort of mental illness, then you end up thinking a lot. You might enter some kind of mental bubble. Where you get very quiet (girl, 16 years old, individual interview 3).

Having started to reach out to family or friends was considered a sign of getting better, and could also facilitate the healing process even further. Social networks were mentioned as tremendously important for getting better, as friends and family helped them break out from isolation, apathy or loneliness. More or less all adolescents also highlighted how merely having someone to talk with, regardless of it being a therapist, family member or a friend, was essential. However, different social arenas hold different functions. Connecting to peers seemed crucial for developing a sense of belongingness and group identity, while connecting with parents provided them with safety needed to encounter new challenges in life. Although it was difficult and for some a huge threshold to open up and let other people know what they were feeling or thinking, starting to reach out and relate seemed to be one of the most influential and strongest positive outcomes, as described by this participant:

For me it has been very helpful that my friends know what I’m struggling with. […] I went to my closest friends about my mental health problems, and told them before I told my parents. They’ve been the best support I ever had (girl, 14 years old, focus group 1).

Establishing or strengthening close relationship with peers is of great importance for the adolescent, although parents are still important figures of attachment. Being able to share with a friend before sharing with parents may be seen as a sign of relational achievements on two important arenas: both relationship with peers, and attachment to parents. In many ways getting better was to recover relationships with family, peers or other significant persons, resulting in a positive spiral towards better life quality and general functioning.

### I’ve started saying yes where I used to say no

In addition to inner processes, a sense of getting better was also something that happened on the outside. At a practical level, the adolescents often stated that a positive outcome was being able to do more things. As one participant put it: “[A good outcome is to] say yes where you’ve used to say no” (boy, 15 years old, individual interview 1). However, this did not mean that a good outcome was saying yes to everything. Rather, a good outcome was to do things they earlier wanted to do but never managed due to their mental health problems. For example, never getting up from bed or spending whole days playing video games or watching TV series, were replaced with going out, meeting friends and showing up at school. In particular, mastering one’s mental health issues and mastering life was an important aspect of this, as exemplified in this excerpt from one interview:

Participant: When you stop doing the things you usually did, […] the last month or week maybe. Take an example, if someone is scared of going to school because they get bullied or have mental health problems. The day you start going to school. Or that day you… dare to… dare to be alone or the day you dare to sleep with the lights off. Those things.

Inquirer: So, getting better is about doing things?

Participant: Yes. I see it that way. […] Yeah, breaking… the voice telling you that you can’t- that you can’t sleep with the lights off because someone will come and get you, or something. You get better when you breach through that… I don’t know… *wall*. Or that inner voice. Instead you go: *Now you have to do it!* That’s getting better. And when you have done it over and over again (boy, 15 years old, individual interview 2).

Working towards small, reasonable sub-goals decided upon in collaboration with the therapist was considered as a cornerstone in the treatment process, and getting better was to start accomplishing these goals. Participants reported having started saying yes and dared to challenge themselves on tasks that earlier seemed unreachable, regardless of this being sleeping with the lights or showing up at school.

### I’ve learned how to cope with life

A positive outcome did not mean that all future problems were solved. On the contrary, to recover from mental health problems was described as a living and forever moving process. Participants stressed that they saw no cure for sadness or fear in life, and keeping on living implied that they would encounter new hindrances. However, from therapy they had learned ways of handling those obstacles. Thus, a positive outcome in psychotherapy was to achieve tools and a state of mind where a passive helplessness was replaced with hope, optimism and agency, as explained by this participant:

Well, when I reflect upon the meaning of getting better, I don’t think it is about getting a hundred percent well. You rather learn new ways of coping with things. So I’m not thinking that now I’ll recover from all my problems. And if I happen to turn out okay in the end, I’ll be positively surprised. But, as I said, I’m going to learn how to… handle things better than what I do right now (girl, 17 years old, individual interview 6).

It appeared that getting better was ongoing, never-ending and even fragile processes, as pointed out in the following to quotes:

So, for me getting better is being able to forget all my problems… or at least many of them. That I’m able to… live in the moment… being present where things happen. That I’m able to enjoy what’s happening around me. But then, it all can fall apart 10 s after. And everything is coming back at me again (girl, 16 years old, individual interview 5).

When I first started here I thought that everything was going to be good. But that’s not the way life is. You’re supposed to be sad sometimes, you won’t be happy all the time. Lower the expectations a little and accept that it’s okay to be sad, to cry, and all that stuff. You need to learn to live with it alone, but I [the therapist] can help you on the way. ‘I can help you to manage it’ (girl, 16 years old, focus group 4).

Indeed, the adolescents stressed that although they felt better at the time being, life was going to get tough sometimes. This did not mean that they had not got better or accomplished a good outcome from psychotherapy. Rather, they had learned that despite the struggle of living, they could handle it or they knew where to seek help. A good outcome was not about living a life without problems. It was rather to have tools to solve them and go on coping with life.

## Discussion

Positive outcome experiences were formulated as having achieved a stronger autonomy and a safer identity. Specifically, being autonomous enough to hope for and to create a better future, being able to interpret own essential emotions and navigate from them, and connecting more deeply with significant others were seen as important. However, being an adolescent fundamentally implied being in constant movement and the process of getting better was not an exception. Some participants underscored its fragile nature. In seconds, their newly established self-esteem could collapse and the hope for a better future would bleach. A risk that a sense of getting better was momentary and potentially shifting always loomed.

The experience of positive outcomes was closely tied to personal development and increased quality of life. This is a form of thinking that falls within a recovery oriented tradition (see e.g. [24] ). In this tradition, recovery is viewed as a deeply personal process of changing one’s attitudes, emotions, goals, skills and roles, and starting living a more satisfying and hopeful life despite one’s limitations caused by illness [25]. This means that recovery is about living as good as possible, as described by our participants in the theme “I’ve learned to cope with challenges in life”. Interestingly, participants also emphasized how recovery is not so much about restoring function or returning to a hypothetic state that once was. Rather, it was a forward-looking process of creating new opportunities in their everyday life.

In relation to the development of outcome monitoring approaches in mental health for adolescents, one important implication of these findings is that outcome measures need to be recovery oriented, sensitive to developmental phases, and focused on the personal goals and values of the unique adolescent. However, most outcome measures are not currently developed in collaboration with service-users, meaning that the definitional power of what constitute “good outcomes” have resided with researchers and policy makers. As a consequence, we would argue that, despite the surge of interest and widespread implementation of routine outcome monitoring seen worldwide, the question of what constitutes a good outcome still for a large part remains disputed. When casting light on the adolescent clients’ own conceptualizations of improvement and outcomes, other ways of resolving some of the major implementation barriers seen across a variety of clinics, especially when it comes to repeated and continuous use of outcome measures (see e.g. [26, 27]), can arise.

For instance, clinicians’ values and attitudes towards outcome monitoring is a documented implementation hindrance [28–30]. Some clinicians argue that existing outcome measures tend to alienate their young clients and does not consider the differences in client needs [29]. Others argue that current measures are too focused on symptom reduction (“curing aspects”), overlooking coping and contextual variables important to track other aspects of improvement [28]. Moreover, similar to our findings, it appears that standardized outcome measures based on symptom improvement match poorly with young service-users’ own conceptualization of improvement and recovery [10]. Hopefully, if outcome measurements were more personally relevant to the young service user, this might also affect the clinician’s perceived clinical usefulness of the scales, and ultimately the young service user’s own motivation to use them.

From the adolescent perspective, how can the ways young service users define “good outcomes” be understood in light of the adolescent’s life and development? Adolescence is a period of life where identity is formed, autonomy gets real and relationships consolidate. Early identifications are integrated into a personal identity, with a new and stronger sense of “who I am”, rather than the echoes from an individual’s identity as a child [31]. In later adolescence, youths start to form more coherent and reflective self-narratives, narratives having a history and a future [32]. Along with this, an overarching attachment organization and approach strategy pointing forward to future intimate relationships emerge from various distinct attachment patterns [33]. Indeed, autonomy and identity are vital developmental achievements in adolescence, which in keeping with our findings also become important goals to achieve in constructive change processes. In summary, successful mental health services seem to help adolescents establish an identity and the autonomy needed to exercise it. Yet, adolescent identity is an ever-changing, unstable process, as identity gets constructed as one moves through life, encountering new experiences, situations and people that affects ones’ self-theory [34].

## Limitations and strengths

Graham [35] claims that the adult world has tended to disempower and infantilize young people. As a society we have failed to take into account their skills and resources, and hindered them in their efforts to put their competences to use. This may be a particularly crucial issue in the field of mental health care, which has traditionally focused more on remediation of illness and symptoms than on the discovery of people’s resources, on setting up interventions through outcomes defined by the health care system rather than basing them in each person’s own goals and aspirations [10, 11, 36]. An important strength of this study is that we develop knowledge on what constitutes good outcomes on the foundation of descriptions from the first-person perspectives of young service users rather than in “professional” and “adult” language. Another potential strength of the study is that we have included adolescents with a broad variety of mental health issues and backgrounds to focus groups and individual interviews discussing shared meanings vis-à-vis the topic of treatment outcomes. This allows us to move beyond the particulars of specific issues, such as for example exposure experience for the adolescent with phobic avoidance, and into what is shared between different ways of suffering.

An important limitation is that all participants had experiences from similar public mental health settings. This may reduce the generalizability of the study findings, as they need to be understood within this Norwegian context, as specified in the methods section. Furthermore, another limitation that we are aware of is that we have not carried out member checking procedures with the participants due to practical reasons. Future studies that do not spread over large geographical areas could beneficially include this strategy. Another limitation is that we have interviewed young people who currently are receiving treatment. It is possible that their views and perspectives differ markedly from persons who have dropped out from treatment, or felt that treatment that they have received was unhelpful. As such it would be of interest with research focus on outcomes from the point of view of young people who have disengaged from mental health care. Moreover, the first person perspective of adolescents as clients in mental health needs more carefully designed studies.

## Conclusion

We have studied the experiences of 22 adolescents aged 14–19 years old to explore what they experienced as “good outcomes” of psychotherapy. We have reported five constituent sub-themes supporting the strengthening of autonomy and identity in the adolescents’ ongoing development, as representing what “good outcomes” mean to them.
